# Severe Adult Kerion Celsi With Rapid Improvement During Systemic Antifungal and Adjunctive Secretome Therapy: A Case Report

**DOI:** 10.7759/cureus.113711

**Published:** 2026-07-31

**Authors:** Dian Andriani Ratna Dewi, Khufitha Tasya, Juliandra Firdaus, Sausan Maulida, Sri Murtiyani

**Affiliations:** 1 Dermatovenereology, The Republic of Indonesia Defense University, Bogor, IDN; 2 Faculty of Medicine, Universitas Kristen Indonesia, DKI Jakarta, IDN; 3 Faculty of Medicine, Yarsi University, DKI Jakarta, IDN; 4 Public Health, Universitas Padjadjaran, Bandung, IDN; 5 Microbiology, Rumah Sakit Umum Pusat (RSUP) Persahabatan, DKI Jakarta, IDN

**Keywords:** adult tinea capitis, cat exposure, inflammatory dermatophyte infection, kerion celsi, microsporum canis, regenerative dermatology, secretome therapy

## Abstract

Kerion celsi is a severe inflammatory form of tinea capitis that is uncommon in adults and may mimic bacterial furunculosis, abscess, or cellulitis, resulting in delayed diagnosis and antibiotic-only treatment. We report a 48-year-old woman with a one-month history of a painful, purulent, crusted lesion on the left temporal and retroauricular scalp, localized alopecia, and ipsilateral periorbital edema. She reported close exposure to 10 cats daily and did not improve after treatment for presumed furunculosis. Potassium hydroxide examination demonstrated fungal elements. Fungal culture and lactophenol cotton blue morphology were consistent with Microsporum canis. Histopathology demonstrated dense suppurative follicular and perifollicular inflammation, follicular destruction, and granulomatous inflammation; fungal organisms were not identified on hematoxylin and eosin staining. The patient received oral griseofulvin, topical miconazole, structured wound care, empirical ciprofloxacin for clinically suspected secondary bacterial infection, and adjunctive intramuscular administration of a human umbilical cord-derived mesenchymal stromal cell conditioned-medium secretome preparation. Periorbital edema markedly decreased by day 2. By day 14, lesion dimensions remained approximately 8 × 5 cm, while pain and pruritus had decreased from 6/10 to 0/10 and exudation, pustulation, crusting, erythema, and scalp edema were substantially reduced. This improvement was temporally associated with the combined regimen. Because several interventions were initiated concurrently, the specific contribution of secretome could not be determined. Systemic antifungal therapy remains the cornerstone of treatment, and the safety and efficacy of secretome in active inflammatory dermatophyte infection require further study.

## Introduction

Tinea capitis is a dermatophyte infection involving the scalp, hair shaft, and hair follicles. Although it is predominantly seen in children, adult tinea capitis is increasingly recognized and may occur in association with animal exposure, household transmission, postmenopausal status, immunosuppression, diabetes mellitus, or delayed diagnosis [1-4].

Kerion Celsi represents the severe inflammatory end of the tinea capitis spectrum. It is characterized by painful boggy inflammatory plaques or nodules, pustules, crusting, purulent exudate, alopecia, and sometimes regional lymphadenopathy or surrounding soft-tissue edema. Severe inflammation may damage hair follicles, resulting in permanent cicatricial alopecia, particularly when diagnosis and systemic antifungal therapy are delayed [1,2,4-6].

Adult Kerion Celsi is diagnostically challenging because suppuration, crusting, tenderness, and edema may lead clinicians to suspect bacterial furunculosis, abscess, carbuncle, or cellulitis. Several reports have shown that kerion may initially be misdiagnosed as a bacterial infection, leading to repeated antibiotic treatment, delayed antifungal therapy, and potential complications [6-8].

Secretome therapy, particularly mesenchymal stromal/stem cell-derived conditioned medium or extracellular vesicle-rich preparations, has been investigated as a cell-free regenerative approach in cutaneous wound healing. Its proposed benefits include modulation of inflammation, promotion of angiogenesis, enhancement of epithelial repair, and support of tissue remodeling [9-11]. However, its role in inflammatory dermatophyte infections such as Kerion Celsi has not been established.

We report a severe adult case of kerion celsi with mycological findings morphologically consistent with Microsporum canis, high-intensity feline exposure, previous treatment failure under a diagnosis of furunculosis, and ipsilateral periorbital edema. Early clinical improvement occurred during combined systemic and topical antifungal therapy, structured wound care, empirical antibiotic treatment, and adjunctive intramuscular administration of a cell-free, human umbilical cord-derived mesenchymal stromal cell conditioned-medium preparation. Because these interventions were initiated concurrently, this temporal observation is presented as hypothesis-generating and does not establish an independent effect of secretome.

## Case presentation

A 48-year-old woman presented to a dermatology clinic with a one-month history of scalp lesions near the left ear and was evaluated by a dermatologist. She reported no known chronic medical conditions, including diabetes mellitus, no known immunocompromising condition, and no regular use of medications, systemic corticosteroids, or other immunosuppressive agents. The lesions initially appeared as acne-like papules, were repeatedly scratched, and gradually increased in number. Three weeks before presentation, after the scalp lesions had already developed, she underwent a keratin-based hair treatment. Because the product formulation and procedural details were unavailable and no pre- and post-procedure assessment was performed, its contribution to subsequent worsening could not be determined; the procedure is reported only as a temporal event. The patient owned 10 cats, frequently slept with them, and reported that one cat had visible hair loss. The cat had not been examined by a veterinarian, and no veterinary or mycological assessment was performed; therefore, a direct zoonotic source could not be microbiologically confirmed. No household members reported similar skin or scalp symptoms. Two weeks before the dermatology consultation, she had been diagnosed with furunculosis and treated with cefixime, cetirizine, and an unspecified topical medication without improvement. Persistent purulent discharge, crusting, tenderness, and localized hair loss prompted reconsideration of the initial diagnosis.

**Figure 1:**
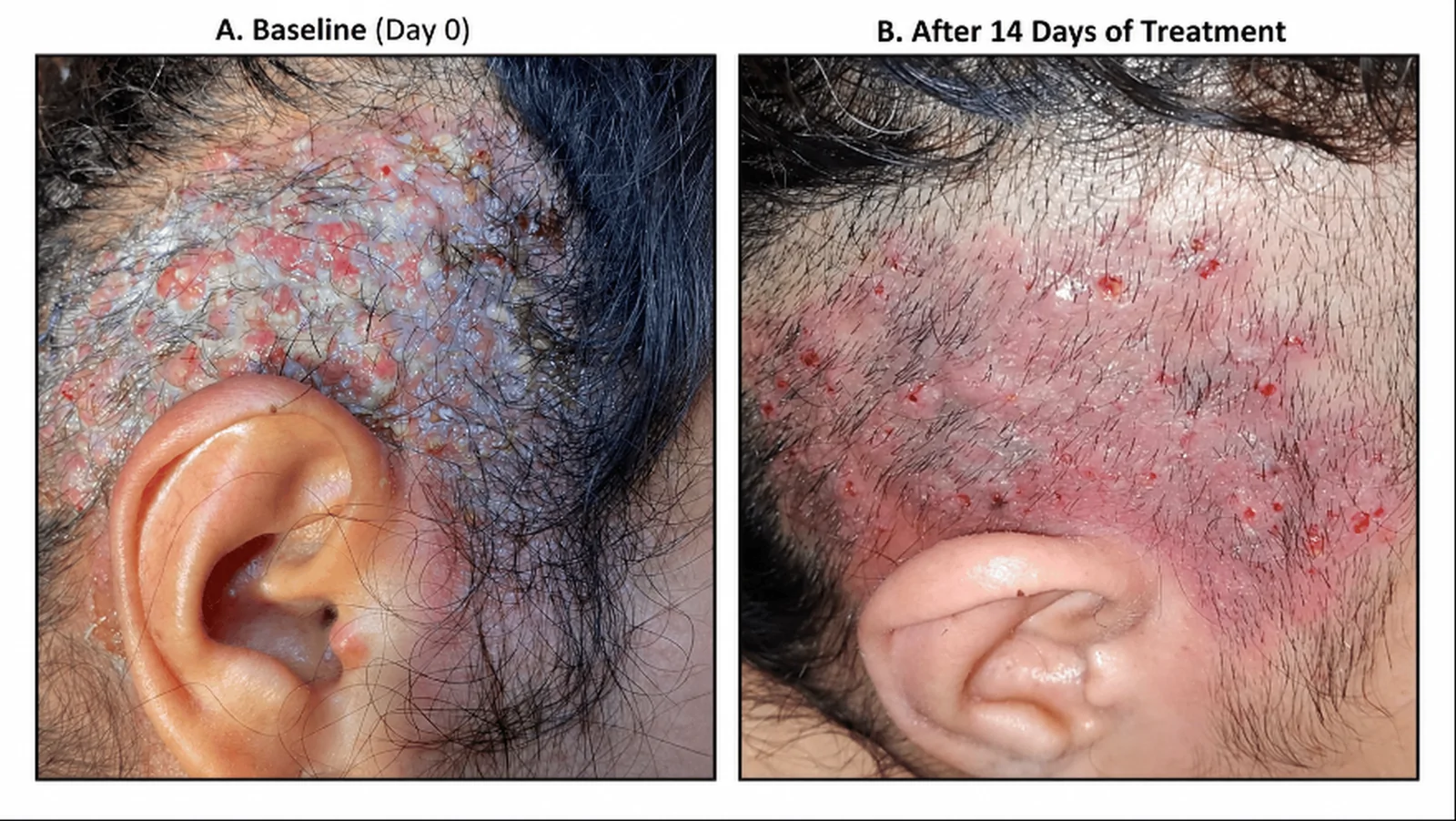
Clinical course of the scalp lesion. (A) At baseline, an 8 × 5 cm erythematous, edematous, pustular, crusted, purulent, and alopecic plaque involved the left temporal and retroauricular scalp. (B) At day 14, lesion dimensions remained approximately 8 × 5 cm, while exudation, pustulation, crusting, erythema, and edema had substantially decreased.

**Figure 2:**
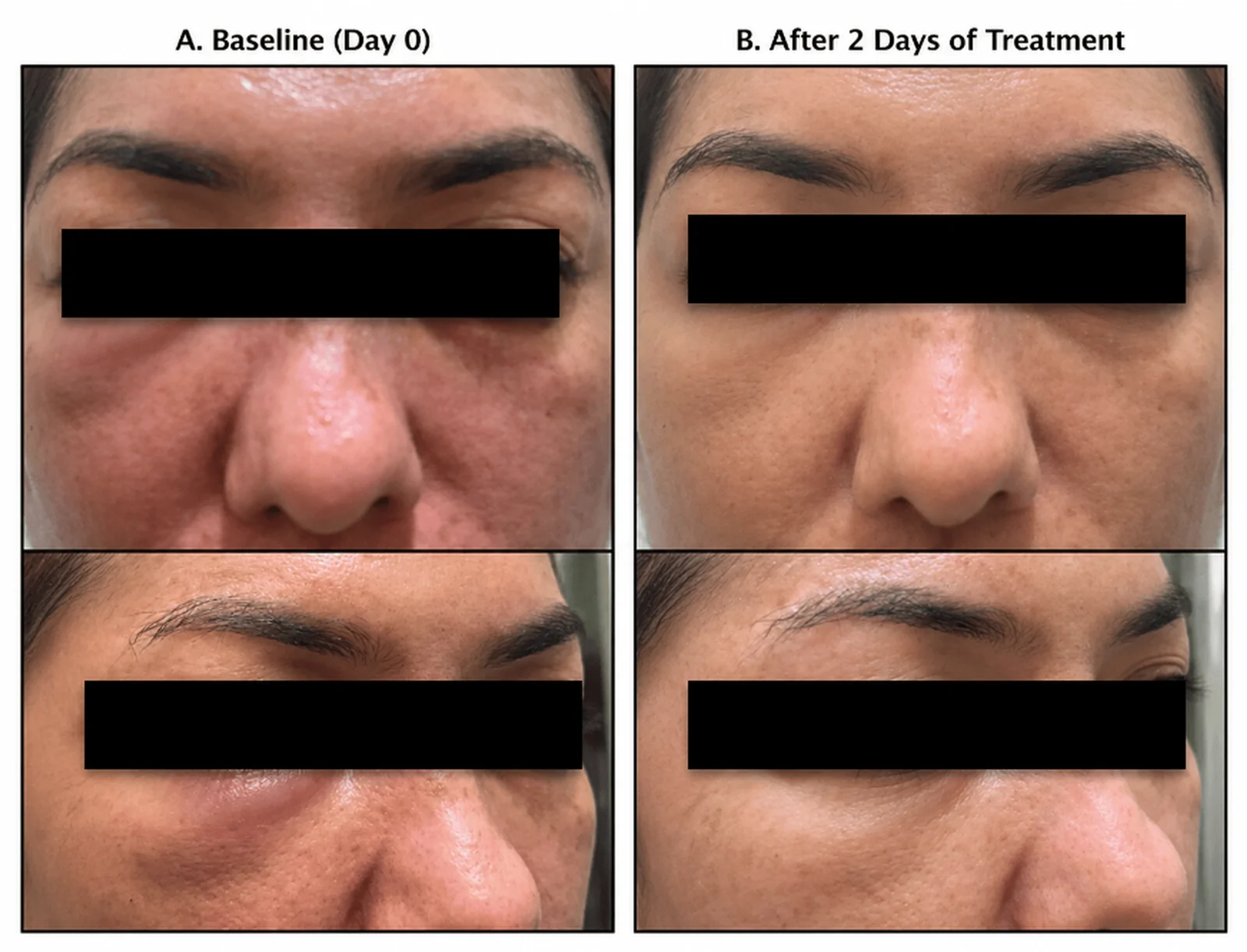
Early improvement of ipsilateral periorbital edema. (A) Ipsilateral periorbital edema at baseline. (B) Marked reduction in periorbital edema at day 2. Serial photographs were obtained using the same camera, distance, angle, lighting conditions, and patient position to facilitate clinical comparison. Written informed consent was obtained for publication of the clinical images.

At baseline, dermatological examination revealed a single confluent erythematous, edematous, pustular, crusted, and alopecic plaque measuring approximately 8 × 5 cm over the left temporal and retroauricular scalp. Severe exudation was present, characterized by spontaneous and profuse purulent discharge from multiple follicular openings, producing a honeycomb appearance consistent with the clinical kerion sign. Pain and pruritus were each rated 6/10 (Figure 1). Ipsilateral facial and periorbital edema and retroauricular lymphadenopathy were also noted (Figure 2).

The patient denied blurred vision, diplopia, ocular pain, pain during eye movement, subjective proptosis, fever, generalized weakness, or deterioration in her general condition. No clinical features of orbital involvement or systemic toxicity were identified. In the absence of visual symptoms, painful or restricted eye movement, proptosis, fever, or systemic deterioration, urgent orbital imaging and emergency ophthalmological evaluation were not pursued. The differential diagnoses included furunculosis, bacterial abscess, carbuncle, bacterial cellulitis, dissecting cellulitis of the scalp, folliculitis decalvans, erosive pustular dermatosis, and inflammatory tinea capitis presenting as kerion celsi.

Diagnostic assessment

Seven days after the final dose of cefixime and before the initiation of griseofulvin, topical miconazole, ciprofloxacin, wound care, or secretome administration, scalp scales and affected hairs were collected for direct potassium hydroxide (KOH) examination and fungal culture. Lactophenol cotton blue microscopy was subsequently performed on the cultured colonies. A tissue specimen was obtained separately for histopathological examination.

**Figure 3:**
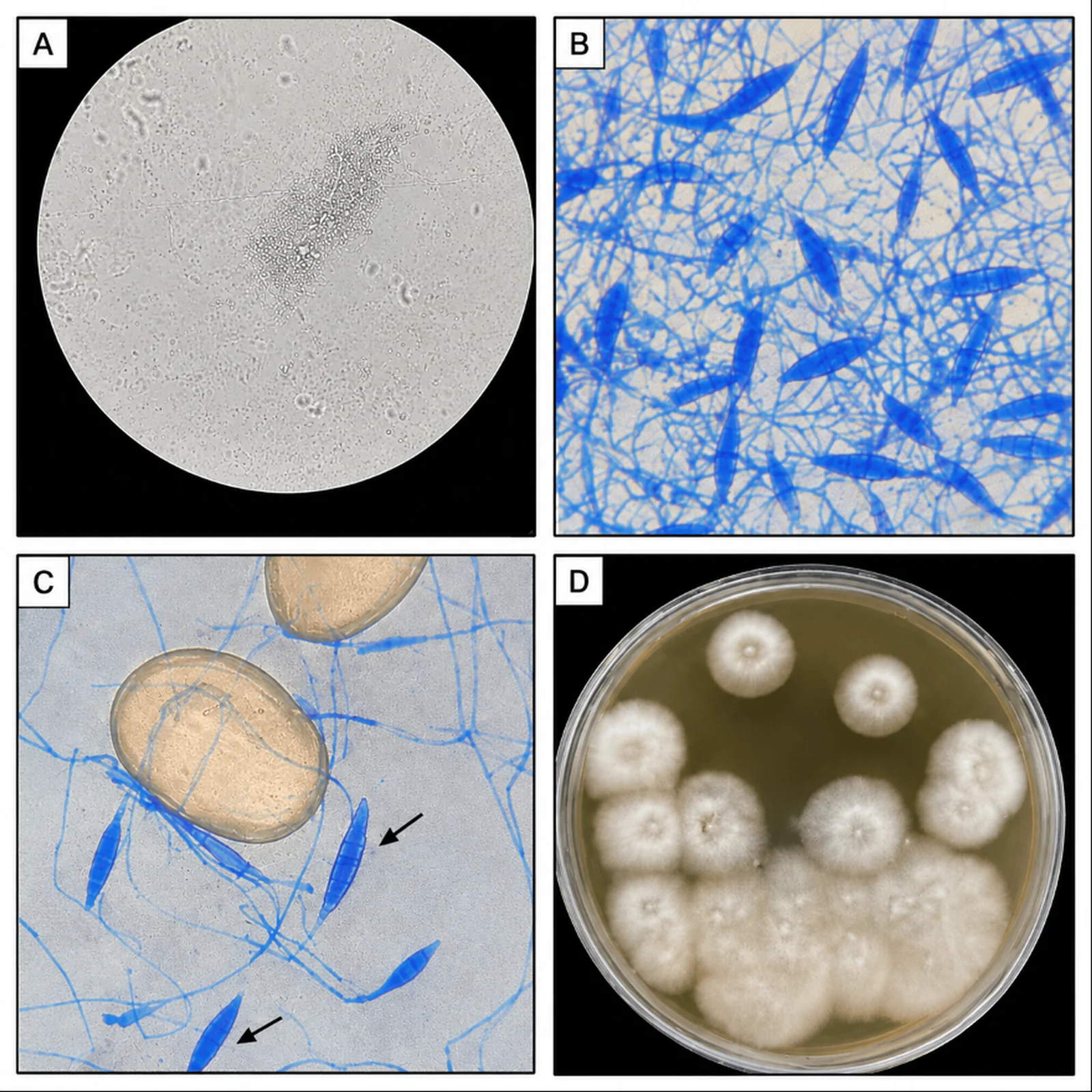
Mycological findings morphologically consistent with Microsporum canis. (A) Direct KOH examination of scales and affected hairs demonstrated fungal elements compatible with dermatophyte infection. (B) Lactophenol cotton blue examination of cultured colonies demonstrated multiple spindle-shaped, rough-walled, multiseptate macroconidia. (C) Higher-power appearance of representative macroconidia (arrows). (D) Fungal culture produced white, cottony colonies. Collectively, the macroscopic and microscopic morphology was consistent with Microsporum canis. No molecular or other confirmatory identification method was performed.

Direct KOH examination demonstrated fungal elements consistent with dermatophyte infection. Fungal culture produced white, cottony colonies. Lactophenol cotton blue staining demonstrated spindle-shaped, rough-walled, multiseptate macroconidia morphologically consistent with Microsporum canis (Figure 3). Species identification was based on macroscopic colony characteristics and microscopic morphology; no molecular or other confirmatory identification method was performed.

**Figure 4:**
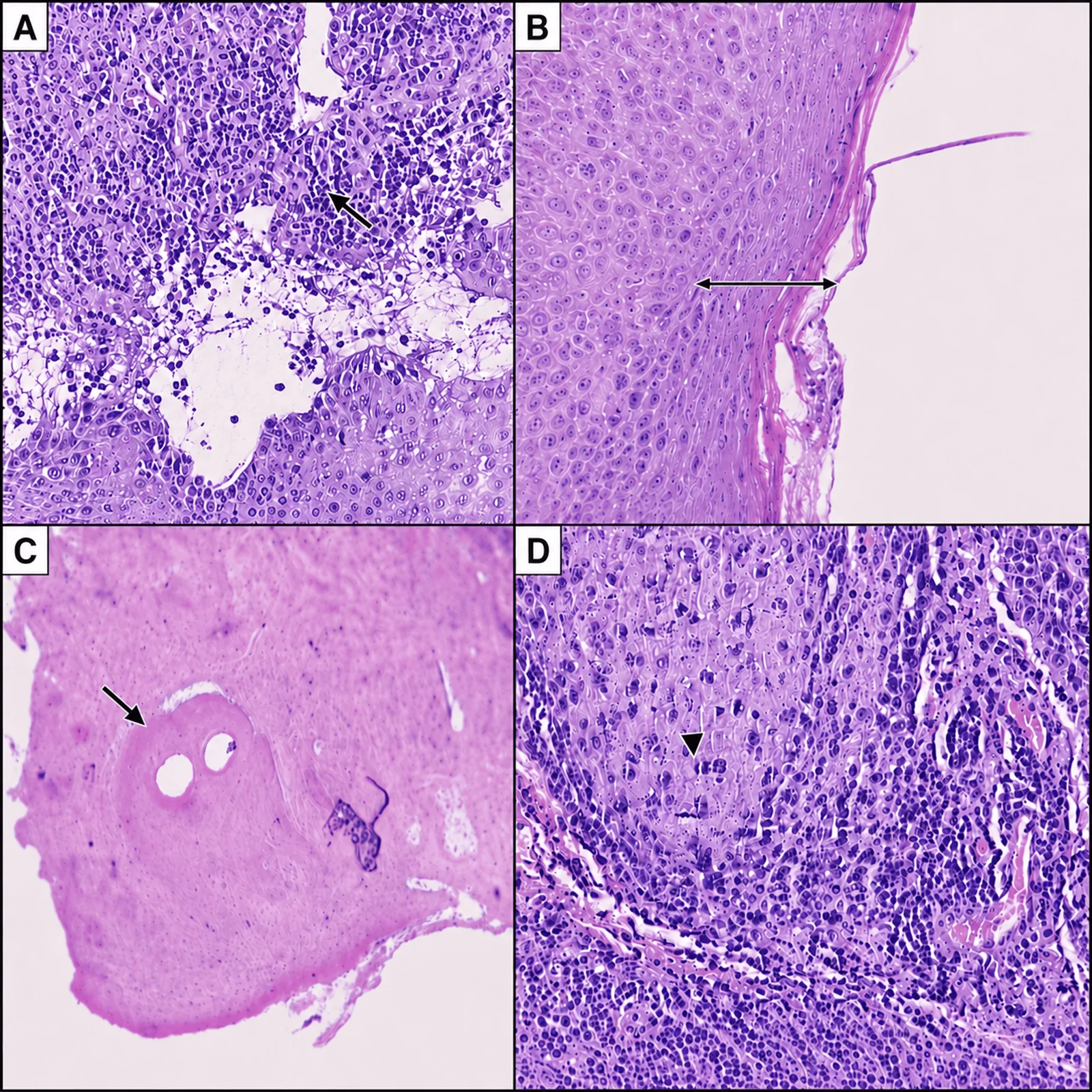
Histopathological findings of Kerion Celsi. Hematoxylin and eosin-stained sections demonstrate (A) dense suppurative follicular and perifollicular inflammation (arrow), (B) epidermal thickening (double-headed arrow), (C) follicular destruction (arrow), and (D) granulomatous perifollicular inflammation with multinucleated giant cells (arrowhead). Fungal organisms were not identified on routine hematoxylin and eosin staining; periodic acid-Schiff and Grocott methenamine silver stains were not performed.

Histopathological examination demonstrated dense suppurative follicular and perifollicular inflammation, epidermal thickening, follicular destruction, and granulomatous inflammation comprising a mixed inflammatory-cell infiltrate and multinucleated giant cells. These findings supported a severe folliculocentric and perifollicular inflammatory process consistent with kerion celsi. Fungal organisms were not identified in the examined hematoxylin and eosin-stained sections, and periodic acid-Schiff and Grocott methenamine silver staining were not performed. Therefore, histopathology supported the inflammatory process, whereas the etiological diagnosis was established through KOH examination and fungal culture (Figure 4).

Therapeutic intervention

The patient weighed 75 kg. Pretreatment laboratory testing showed leukocytosis (13.1 × 10³/µL) with relative neutrophilia (75.1%). Liver-function parameters, including AST (28 U/L), ALT (30 U/L), GGT (35 U/L), and total bilirubin (0.53 mg/dL), were within the laboratory reference ranges.

Microsized oral griseofulvin was prescribed at 500 mg twice daily (total daily dose of 1,000 mg; approximately 13.3 mg/kg/day), with an intended treatment duration of eight weeks. At the last documented assessment on day 14, the patient had completed 14 days of griseofulvin therapy, remained on treatment, and reported taking all prescribed doses without interruption. Monitoring consisted of pretreatment liver-function testing and clinical assessment of adherence and tolerability. Repeat liver-function testing had not yet been performed by day 14. Therefore, completion of the intended eight-week course, longer-term adherence, and longitudinal biochemical safety could not be assessed in this report.

Topical miconazole 2% was applied twice daily for an intended duration of six to eight weeks and remained ongoing at the day-14 assessment. Crusts and purulent exudate were gently removed, and the scalp was cleansed once daily with sterile 0.9% sodium chloride solution, followed by a honey-impregnated dressing for seven days. Chlorpheniramine maleate 4 mg was administered twice daily as needed for pruritus, and elemental zinc 20 mg was administered twice daily for 10 days.

In the context of profuse purulent exudation, the pretreatment leukocytosis and relative neutrophilia raised clinical concern for possible secondary bacterial infection. Oral ciprofloxacin 500 mg twice daily was therefore administered empirically for five days. However, the patient was afebrile and had no evidence of systemic toxicity. No bacterial culture or antimicrobial-susceptibility testing was performed; therefore, bacterial superinfection remained clinically suspected but was not microbiologically confirmed. The leukocytosis, relative neutrophilia, and purulent discharge could also have reflected the marked inflammatory response associated with kerion celsi.

Under the care of the treating dermatologist and after treatment-specific written informed consent was obtained, the patient received a secretome preparation derived from the conditioned medium of human umbilical cord-derived mesenchymal stromal cells, supplied by Stem Cell and Cancer Research Indonesia (SCCR Indonesia), Semarang, Indonesia. Secretome was administered as a non-standard adjunctive intervention during individualized clinical care and was not administered under a prospective research protocol.

According to documentation reviewed by the authors, the preparation (batch number 7D1412802104B) was manufactured at a facility operating under cGMP standards. A batch-specific certificate of analysis and complete quantitative composition, standardized potency, sterility, and quality-control documentation were not available to the authors; therefore, these product characteristics could not be independently assessed. Facility-level cGMP certification does not constitute product-specific regulatory approval or evidence of clinical efficacy in kerion celsi.

Under sterile conditions, secretome was administered exclusively by intramuscular injection into the deltoid muscle at a volume of 1.5 mL once daily for five consecutive days. The first injection was given on the same day that systemic antifungal therapy and structured wound care were initiated. No secretome was administered topically, intralesionally, or directly to the scalp lesion.

The intramuscular route was selected by the treating clinician as a systemic delivery route for the supplied preparation. Direct application to the scalp was not performed because the preparation had not been formulated or validated for topical application to an actively infected and inflamed lesion. The deltoid was selected as a standard and accessible intramuscular injection site. Neither this route nor the administered dosing regimen has established efficacy for kerion celsi. Secretome was used only as an adjunct and did not replace systemic antifungal therapy.

Safety observation extended from the first secretome injection through the day-14 assessment, including approximately nine days after the final injection. Clinical monitoring included assessment for injection-site pain, erythema, swelling, hypersensitivity reactions, fever, other systemic symptoms, and clinical worsening of the scalp infection. No immediate injection-related reactions or delayed local or systemic adverse events were observed or reported during this period. Adverse events occurring beyond day 14 could not be assessed.

Follow-up and outcomes

The patient reported adherence to the prescribed regimen throughout the documented 14-day period. No clinical symptoms suggestive of griseofulvin intolerance were reported; however, biochemical safety was not reassessed because repeat liver-function testing had not yet been performed.

Ipsilateral periorbital edema markedly decreased by day 2. At day 14, lesion dimensions remained approximately 8 × 5 cm; however, pain and pruritus had decreased from 6/10 at baseline to 0/10. Purulent exudation, pustulation, crusting, erythema, and scalp edema were also substantially reduced (Table 1). The serial photographs were obtained using consistent acquisition conditions and were interpreted together with the documented lesion dimensions and symptom scores.

**Table 1:** Clinical Timeline hUC-MSC: human umbilical cord-derived mesenchymal stromal cell; IM: intramuscular; QC: quality control.

Time point	Clinical findings/events	Diagnostic or therapeutic interpretation
One month before presentation	Acne-like papules appeared on the scalp near the left ear, were repeatedly scratched, and gradually increased in number.	Early inflammatory scalp eruption; dermatophyte infection had not yet been recognized.
Three weeks before presentation	After the lesions had already developed, the patient underwent a keratin-based hair treatment.	The procedure was recorded as a temporal event. Its contribution to subsequent worsening could not be determined.
Two weeks before presentation	The patient was diagnosed with furunculosis and treated with cefixime, cetirizine, and an unspecified topical medication.	Lack of improvement following antibiotic-based treatment prompted reconsideration of the initial diagnosis.
Ten days before presentation	The lesions became increasingly inflamed and purulent.	The suppurative presentation increased the likelihood of misclassification as bacterial furunculosis, abscess, or cellulitis.
Day 0 / May 17, 2026	Examination demonstrated an 8 × 5 cm crusted, pustular, purulent, and alopecic plaque over the left temporal and retroauricular scalp, with ipsilateral facial and periorbital edema and retroauricular lymphadenopathy. Seven days after the final cefixime dose and before new treatment, specimens were obtained for direct KOH examination, fungal culture, and histopathology; lactophenol cotton blue microscopy was subsequently performed on the cultured colonies.	Kerion celsi was suspected. Systemic and topical antifungal therapy, structured wound care, empirical ciprofloxacin, and adjunctive secretome were initiated.
Days 0-4	Cell-free hUC-MSC conditioned-medium secretome (SCCR Indonesia; batch 7D1412802104B) was administered as a non-standard adjunct at 1.5 mL by deltoid intramuscular injection once daily for five consecutive days after treatment-specific written consent. No topical or intralesional secretome was administered.	Secretome was administered as an adjunct and did not replace systemic antifungal treatment.
Day 2	Ipsilateral periorbital edema markedly decreased.	Early clinical improvement occurred during the combined therapeutic regimen; the effect of any individual intervention could not be determined.
Day 14	Lesion dimensions remained approximately 8 × 5 cm. Pain and pruritus decreased from 6/10 to 0/10, with substantial reductions in inflammation, exudation, pustulation, crusting, erythema, and edema. No immediate or delayed secretome-related adverse event was observed or reported through day 14.	Clinical improvement was temporally associated with combined treatment. The independent contribution of adjunctive secretome could not be established.

The observed improvement was temporally associated with the combined regimen of systemic and topical antifungal therapy, structured wound care, short-course empirical antibiotic therapy, and adjunctive secretome administration. Because these interventions were initiated concurrently, the specific contribution of secretome could not be determined, and no causal relationship between secretome administration and the clinical improvement can be established.

The patient was advised to avoid scratching or touching the lesion and to avoid sharing combs, towels, bedding, or head coverings. She was also advised to have her cats examined by a veterinarian to identify and treat a potential source of reinfection. Written informed consent was obtained for the adjunctive secretome intervention and for publication of the case report and accompanying clinical images.

## Discussion

This report describes severe adult kerion celsi with mycological findings morphologically consistent with Microsporum canis, high-intensity feline exposure, previous non-response to antibiotic treatment, and ipsilateral periorbital edema. The case highlights the potential for inflammatory tinea capitis to mimic a bacterial scalp infection and documents the early clinical course during combined treatment.

The initial diagnosis of furunculosis was clinically understandable because purulence, crusting, tenderness, and swelling often point to a bacterial infection. However, some features suggested inflammatory tinea capitis instead: the long-lasting nature, hair loss in one area, failure to improve with antibiotics, a very inflamed patch of scalp, and close contact with many cats. Tinea capitis in adults is often overlooked because it is more commonly seen in children. This bias can delay fungal testing and the initiation of antifungal treatment [1-4].

The exposure history was clinically important. Zoophilic dermatophytes, particularly Microsporum canis, are associated with cats and dogs and may produce marked inflammatory disease. In a patient with suppurative scalp lesions and close feline contact, zoonotic dermatophytosis should be considered early. In this case, one cat reportedly had visible hair loss; however, no veterinary examination or fungal testing was performed. Feline-to-human transmission was therefore suspected on epidemiological grounds but was not microbiologically confirmed. Veterinary assessment and source-control measures were recommended to reduce the risk of reinfection and household spread [12-15].

Ipsilateral periorbital edema raised concern for extension of inflammation into adjacent soft tissue and for possible preseptal or orbital infection. However, the patient was afebrile and had no visual disturbance, diplopia, ocular pain, pain with eye movement, subjective proptosis, systemic toxicity, or clinical deterioration. Purulence and edema can occur as part of the marked inflammatory response of kerion celsi, although secondary bacterial infection could not be excluded because no bacterial culture was obtained. Antibiotic therapy should remain adjunctive when bacterial superinfection is suspected and must not replace systemic antifungal treatment [1,6-8].

Direct KOH examination and fungal culture are important in the etiological assessment of suspected tinea capitis. In this case, KOH examination demonstrated fungal elements, while macroscopic colony characteristics and lactophenol cotton blue morphology were consistent with Microsporum canis. Because no molecular or other confirmatory identification method was performed, species attribution remained morphology-based. Histopathology characterized the severe folliculocentric inflammatory response but did not establish fungal etiology because fungal organisms were not identified on hematoxylin and eosin staining and PAS and GMS stains were not performed.

Trichoscopy may provide a rapid, noninvasive adjunct in patients with suspected tinea capitis. Reported findings include comma hairs, corkscrew hairs, Morse-code hairs, zigzag hairs, broken hairs, and black dots. Nevertheless, KOH examination and fungal culture remain necessary for etiological confirmation [16].

Systemic antifungal treatment is essential for tinea capitis because topical antifungal agents alone do not adequately penetrate the infected hair shaft and follicles. Griseofulvin remains relevant when Microsporum infection is suspected. Terbinafine, itraconazole, and fluconazole are other options, depending on the species, availability, patient needs, drug interactions, and response [1,2,4,6,17]. For this patient, wound care was used to remove crusts and pus, improve hygiene, and make monitoring easier. However, wound care and cleaning are only supportive; systemic antifungal therapy is the main treatment.

Interpretation of adjunctive secretome use during combined therapy

Adjunctive treatment included a cell-free secretome preparation derived from human umbilical cord mesenchymal stromal cell conditioned medium and administered intramuscularly during combined therapy.

For descriptive clinical context, selected previously reported Microsporum canis or cat-associated kerion celsi/tinea capitis cases are summarized in Table 2.

**Table 2:** Contextual Summary of Selected Reported Microsporum canis or Cat-Associated Kerion Celsi/Tinea Capitis Cases The selected reports are summarized solely to provide descriptive clinical context. Differences in baseline severity, treatment, outcome definitions, and follow-up schedules preclude comparison of treatment efficacy or recovery time. Secretome administration was not described in the previous reports; the absence of such reporting should not be interpreted as evidence of non-use or as a comparator for clinical efficacy.

Report	Clinical context	Reported management	Reported outcome/follow-up
Mazlim and Muthupalaniappen [7]	Adult patient with cat exposure and kerion initially treated as a bacterial infection.	Diagnostic reassessment followed by systemic antifungal therapy.	Clinical improvement was reported, but day-2 and day-14 outcomes were not presented in a format comparable with the present case.
Feetham and Sargant [8]	Kerion presenting as a scalp infection initially considered bacterial.	Systemic antifungal therapy after diagnostic recognition.	The report emphasized diagnostic recognition; comparable serial early-outcome measurements were not reported.
Capoor et al. [12]	Familial cluster of M. canis tinea capitis in a non-endemic setting.	Systemic antifungal treatment and household assessment.	Household transmission and species identification were emphasized; the follow-up schedule was not directly comparable.
Shimizu et al. [13]	Inflammatory tinea capitis associated with transmission of M. canis from domestic cats.	Antifungal therapy and source-control measures.	The report emphasized veterinary assessment and source control; comparable day-2 and day-14 outcomes were not reported.
Present case	A 48-year-old woman with exposure to 10 cats, severe purulent kerion, periorbital edema, and mycological findings morphologically consistent with M. canis.	Systemic and topical antifungal therapy, structured wound care, empirical ciprofloxacin for clinically suspected bacterial superinfection, and adjunctive intramuscular secretome.	Periorbital edema markedly decreased by day 2. By day 14, pain and pruritus decreased from 6/10 to 0/10 and exudation, pustulation, crusting, erythema, and edema were substantially reduced; lesion dimensions remained approximately 8 × 5 cm.

In this patient, ipsilateral periorbital edema markedly decreased by day 2, followed by reductions in scalp inflammation, crusting, pustulation, exudation, pain, and pruritus by day 14. These changes occurred after the concurrent initiation of systemic and topical antifungal therapy, removal of crusts and purulent material, structured wound care, empirical antibiotic treatment, and adjunctive secretome administration and may reflect the expected response after initiation of effective antifungal treatment and correction of the initial diagnosis. Although mesenchymal stromal cell-derived secretomes have demonstrated potential immunomodulatory, angiogenic, and tissue-repair properties in preclinical studies [9-11], these findings cannot be directly extrapolated to active kerion celsi. Accordingly, the observed response was temporally associated with the overall combined regimen and does not establish that secretome accelerated healing or provided additional clinical benefit.

Limitations

This case report has several limitations. Its uncontrolled design and the concurrent initiation of multiple interventions prevented isolation of the effect of any individual treatment. The 14-day follow-up allowed assessment of the early clinical response but was insufficient to determine completion of the planned antifungal course, mycological cure, definitive hair regrowth, recurrence, scarring alopecia, or adverse events occurring after day 14. Although serial photographs were obtained using consistent acquisition conditions and lesion dimensions and pain/pruritus scores were documented, changes in exudation, pustulation, crusting, erythema, and edema were assessed qualitatively because no validated kerion-specific severity instrument is currently established.

Additional diagnostic limitations included the absence of bacterial culture and antimicrobial-susceptibility testing, molecular confirmation of species identification, and PAS or GMS staining. Product-related limitations included the absence of a batch-specific certificate of analysis and complete composition, potency, sterility, and quality-control documentation, which precluded independent assessment of the batch-specific product characteristics and product-specific regulatory status.

## Conclusions

Severe kerion celsi can occur in adults and may mimic bacterial furunculosis, abscess, or cellulitis. In this patient, high-intensity feline exposure, localized alopecia, antibiotic non-response, positive KOH examination, supportive histopathology, and culture morphology consistent with Microsporum canis supported the diagnosis. Early improvement occurred during combined systemic and topical antifungal therapy, structured wound care, empirical antibiotic treatment, and adjunctive secretome administration and cannot be attributed to secretome. Systemic antifungal therapy remains the cornerstone of management. This observation is hypothesis-generating, and appropriately controlled studies are required to evaluate the safety and potential adjunctive role of secretome.
